# Clinical characteristics of oral lichen planus and its causal context with dental restorative materials and oral health-related quality of life

**DOI:** 10.1186/s12903-021-01622-z

**Published:** 2021-05-15

**Authors:** Linda Daume, Constance Kreis, Lauren Bohner, Susanne Jung, Johannes Kleinheinz

**Affiliations:** Department of Cranio-Maxillofacial Surgery, University Hospital Muenster, Albert-Schweitzer-Campus 1, 48149 Muenster, Germany

**Keywords:** Oral lichen planus, Dental materials, Metals, Amalgam, OHIP

## Abstract

**Background:**

The aim of this study was to investigate the influence of clinical characteristics and dental restorative materials on oral health-related quality of life in patients with oral lichen planus. In particular, the influences of amalgam and metals were investigated.

**Methods:**

A total of 112 patients with clinical and histological features of oral lichen planus from the Department of Cranio-Maxillofacial Surgery at the University Hospital of Münster participated in this prospective study. Clinical parameters of oral lichen planus and the dental restorative materials used were evaluated. Oral health-related quality of life was investigated by using the short form of the German version of the Oral Health Impact Profile (OHIP-14). In addition, physical pain was rated on a visual analogue scale.

**Results:**

The average OHIP-14 score was 13.54. A high correlation was seen between OHIP and pain. Likewise, higher OHIP-values were seen for male patients, and such as for those patients with non-reticular forms of oral lichen planus (OLP). A local form of OLP is more often seen on female patients, such as with the presence of reticular lichen. In regard to the restorations, the presence of composite restorations is correlated with a local lichen, whereas the presence of gold restorations is often seen with a generalized lichen. Furthermore, the grading of strength of association between mucosal lesion and amalgam/metal was tested. No significant differences revealed the analysis of the relationship between gender, clinical form of OLP, age, and presentation form between the 4 gradings of Thornhill.

**Conclusions:**

The oral health-related quality of life is significantly limited in patients with oral lichen planus. But these OHIP scores are not influenced by the restorative materials. Here, pain severity is the most important aspect. We found no statistical differences in the clinical parameters between patients with amalgam or metal restoration and patients without these restorations. It is not necessary to replace amalgam fillings that are not in direct contact with mucosal surfaces.

**Supplementary Information:**

The online version contains supplementary material available at 10.1186/s12903-021-01622-z.

## Introduction

Oral lichen planus (OLP) is a chronic autoimmune, inflammatory-like mucocutaneous disease. Chronical oral inflammations affect oral and general conditions. Recently the correlation between oral health and systemic diseases or heritable diseases have been discussed [1, 2].

OLP is more prevalent in females than in males with a ratio of approximately 2:1, mostly affecting the middle-aged population. Giuliani et al. showed on a recently systematic review a worldwide prevalence of 1.01% with being more prevalent in women than in men [3]. The risk of malignant transformation is 1.2% [4].

The etiopathogenesis is still unknown. Genetic, infectious, pharmacological, immunological, neurological, and psychological causes were discussed as a T-cell mediated disease in which the auto-cytotoxic CD8+ T-cells trigger apoptosis of the basal cells of the oral epithelium is more secured [5].

Some authors suggest that metal and amalgam restorations may induce OLP or oral lichenoid reactions. The definition of the terms "oral lichen planus" (OLP) and "oral lichenoid reaction" (OLR) is unclear [6]. The oral lichenoid reaction can be attributed to a reaction to a corresponding aetiology, for example, medications or dental agents such as amalgam [6–8]. The exact relationship is unknown and the role of amalgam remains controversial [9]. Some studies have shown healing after removal of dental materials [10]. Clinically and histologically, OLP and OLR cannot be differentiated [8, 11].

According to the clinical classification according to Andreasen reticular, papular, plaque, atrophic, erosive-ulcerative, and bullous forms of OLP are distinguished [12]. Reticular OLP is the most common clinical form. The symptoms are variable,about 2/3 of the patients describe a burning sensation and pain in the area of the oral mucosa [13]. At present there is no curative treatment for OLP, the therapy is purely symptomatic [14]. Since it is a chronic disease with recurrent symptoms and lesions, many of the affected patients not only have significant oral limitations but have social and psychological impairments. Oral health-related quality of life (OHRQoL) is a useful tool for measuring the impact of oral diseases and associated treatment based on patients’ perceptions. This subjective perception is important for the assessment of treatment needs, clinical situation, and therapy planning [15]. In this study we evaluated the alterations on the quality of life of patients with OLP. OHRQoL can be used in many applications. For example, the impact of diabetes or gingivitis on oral health-related quality of life was evaluated in the literature [16, 17].

### Objectives

In order to clarify the etiological factors related to OLP, the present study purposed to investigate the relationship between dental restorative materials and OLP.

## Material and methods

One hundred twelve patients previously diagnosed at the Department of Oral and Maxillofacial Surgery, Hospital University Münster were included in this study. The study was approved by the Ethics Committee of the Medical Association of Westphalia-Lippe and the Westphalian Wilhelms University Münster (Ref.No. 2019-033.f-S). All patients were previously clinically examined. Clinical signs were documented with photos and diagnosis was confirmed with a biopsy. We excluded patients with other confirmed oral mucosal diseases and patients after radiotherapy. The different dental materials were documented. The following materials were available: composite, amalgam, ceramic, gold, and non-precious metal. The association of OLP with metal/amalgam followed the criteria proposed by Thornhill et al. (Table 1) [9]

**Table 1:** Thornhill grading: grading of strength of association between mucosal lesions and amalgam/metal

Type I	No association	No lesions in direct contact with amalgam restorations. Lesions have typical bilaterally symmetrical distribution of oral lichen planus or are restricted to areas of the palate, gingivae, or outer surface of the lip that do not come into contact with the teeth
Type II	Weak association	Some amalgam restorations in contact with affected areas of mucosa. However, < 25% of affected mucosa in direct contact with amalgam restorations. Some amalgam restorations may also be in direct contact with unaffected areas of oral mucosa
Type III	Strong association	> 75% of affected mucosa in direct contact with amalgam restorations. No amalgam restorations in direct contact with unaffected areas of oral mucosa
Type IV	Very strong association	Lesions restricted to but affecting all areas of mucosa in direct contact with amalgam restorations

Patients were divided into two groups according to the clinical presentation of OLP: “reticular OLP” and “non-reticular OLP”. The non-reticular form included all patients with erosive-ulcerative, bullous and atrophic OLP. Papular and plaque OLP did not occur in the patients examined. In addition, a distinction was made between a “local” and a “generalized” presentation form of the disease. If the OLP was visible in more than three sites in the oral cavity, it was defined as a “generalized” presentation form.

Further patients received a questionnaire with open questions and the German version of the OHIP-14 questionnaire (see Additional file 1: OHIP-14 questionnaire) to evaluate subjective Oral Health-Related Quality of Life (OHRQoL) [18]. Additionally, anamnestic data on age and gender were collected. The questionnaires were completed and evaluated anonymously. The OHIP-14 questionnaire contains seven domains of questions (“functional limitation”, “physical pain”, “psychological discomfort”, “physical disability”, “psychological disability”, “social disability”, and “handicap”). The possible answers concerning reduced quality of life are given on a “Likert-type” scale (0 = never, 1 = hardly ever, 2 = occasionally, 3 = often and 4 = very often). A maximum of 56 points can be obtained with 8 points in each subgroup. The essence of the score shows that the higher the overall score, the worse is the OHRQoL. In addition, patients rated current physical pain on a visual analogue scale (VAS) of 0–10. The scale was given as a bar of 10 cm on which the patient marked the intensity of sensation as a distance from the left edge (0 cm = no pain, 10 cm = most pain) [19]. All methods were carried out in accordance with relevant guidelines and regulations.

Statistical analysis was performed with SPSS 22.0 (IBM). First adherence to normality was assessed with Kolmogorov–Smirnov’s test and normality curve. As continuous data adhered to the normal curve (*p* > 0.05), they were described as mean ± standard deviation. Correlation between continuous and categorical data was evaluated using Spearman’s correlation test, whereas correlation between continuous data was assessed by Pearson’s test. The following grading of the degree of correlation was applied: 0.1–0.3 marks a low, 0.3–0.5 a moderate, and > 0.5 a high correlation. After, a multilevel approach was performed to assess the relation between OHIP values and the independent variables “pain”, “gender”, and “lichen presentation”. Logistic regression was applied to analyze the interaction between “presentation form” with “gender”, “reticular lichen”, and the presence of “gold” and “composite” restorations. The pain scale was distributed as dummy variables. A significance level at *p* = 0.05 was considered.

## Results

Twenty-one male and ninety-one female patients with a mean age of 60 ± 10 years were recruited for the study. Significantly more women were examined (*p* < 0.05). The average total OHIP-14 value was 13.54 (± 10.28) points (Table 2). In regard to gender, a difference of 6,54 points was seen between gender, being higher for males in comparison to females.

**Table 2:** Descriptive data

	Mean ± standard deviation	95% Confidence interval	
		Inferior	Superior
Age (years)	60.00 ± 10.07	58.00	61.99
OHIP-values (0–56)	13.54 ± 10.28	11.50	15.58

The most common restorative materials were composite (84.8% of all patients) and ceramics (72.3% of all patients), as described in Table 3. When comparing different materials, there were no statistically significant differences between gender, OLP clinical forms, and OHIP-scores (*p* > 0.05).

**Table 3:** Dental restorative materials assessed in this study

Dental material	Total (n = 112)	%
Composite	95	84.8
Amalgam	55	49.1
Ceramic	81	72.3
Gold	47	42.0
Non-precious metal	69	61.6

Table 4 described the correlation test for OHIP-values. A high positive correlation was found between OHIP and pain (*p* < 0.01), whereas a negative moderate correlation was found for gender (*p* < 0.01) and reticular OLP (*p* = 0.01). In addition, higher OHIP-values were seen for male patients, such as for the non-reticular form of OLP (*p* < 0.05). No correlation was found between OHIP-values and the grading of strength of association between the mucosal lesion and amalgam or metal (Thornhill grading).

**Table 4:** Correlation test for OHIP-values

	Gender	Age	Pain	Reticular lichen	Lichen presentation form	Thornhill Amalgam	Thornhill Metal
Correlation coefficient	-0.41*	0.10	0.62*	− 0.23*	− 0.28	− 0.05	− 0.10
*p* value	< 0.01	0.27	< 0.01	0.01	0.76	0.68	0.27

^*^Means statistical significant difference

Lichen presentation form was positively correlated with gender and reticular lichen (*p* < 0.01) (Table 5). That indicates a local form is often seen in female patients, such as with the presence of reticular OLP. In regard to the restorations, the presence of composite restorations is correlated with a local OLP (*p* = 0.01), whereas the presence of gold restorations is often seen with a generalized OLP (*p* < 0.01). However, these correlations are low and possibly not clinically significant. Furthermore, the grading of strength of association between mucosal lesion and amalgam/metal was tested. Analyzing the relationship between gender, clinical form of OLP, age, and presentation form, no significant differences could be found between the four gradings of Thornhill. We analyzed amalgam and metal in general.

**Table 5:** Correlation test for lichen presentation form

	Gender	Age	Reticular Lichen	Amalgam	Gold	Non-precious metal	Composite	Ceramic	Thornhill Amalgam	Thornhill Metal
Correlation coefficient	0.24*	0.01	0.38*	0.02	− 0.27*	− 0.06	0.22*	− 0.06	− 0.03	0.05
*p* value	< 0.01	0.86	< 0.01	0.79	< 0.01	0.51	0.01	0.50	0.82	0.16

*Means statistical significant difference

### Multilevel analysis

As shown in Table 6, an increase in OHIP values can be explained by 27% by the pain, and only 5% is related to gender. That means pain severity is the most important contributor to the increase of OHIP-values, and severe pain is the most influential to increase OHIP-values.

**Table 6:** Multilevel analysis for OHIP values

Model	R	R square	Adjusted R square	Standard error of the estimate	R Square change	f change	df1	df2	Significance of F-change	Durbin-Watson statistics
1 (pain)	0.53^a^	0.28	0.27	8.60	0.28	21.94	2	109	0.00	2.11
2 (gender)	0.58^b^	0.34	0.32	8.27	0.06	9.90	1	108	0.002	

R, R, correlation coefficient; df, degree of freedom

^a^Factors (konstant): pain

^b^Factoren (konstant): pain, gender

## Discussion

In order to clarify the aetiological factors related to OLP, the present study purposed to investigate the relationship between dental restorative materials and OLP. Differences between patients with and without metal or amalgam were studied. As a clinical consequence, advice on dental sanitation should be given. Furthermore, the hypothesis that individual and intra-oral factors correlate with OHIP-values, needs to be considered. The influence of individual and intra-oral factors on the presentation form of the disease must be analyzed.

Patients had an average age of 60 years and 81.25% were women. In general, more middle-aged women than men are affected. They also achieved significantly higher OHIP values, although the severe generalized cases of lichens of this study occurred more often in men. A Swedish group analyzed the gender-specific incidence of autoimmune diseases from national registers and revealed that the classical view of the female predominance of autoimmune diseases may be far from striking than previously believed [20].

Adverse reactions in the oral cavity due to contact to dental material have been described in numerous studies. The most commonly problems of local exposure to restorative materials are local inflammatory reactions due to toxic irritant or allergic effects [21]. Especially the safety of amalgam has been discussed. The continuous low-level release of mercury of amalgam fillings is concerning. The main concerns relate to the potential toxic effects of mercury and the possibility that mercury may induce adverse immunological reactions [22].

Several studies suggest that dental amalgam fillings and metal restorations may induce oral lichen planus or oral lichenoid reactions in the oral mucosa in susceptible patients. Skin patch test studies investigated the contact sensitivity response to dental materials of OLP patients. Several studies produced conflicting results with a span of 8 to 92% of OLP patients being positive [9, 21]. A review of Issa et al. concluded that the evidence from observational studies suggests that patch testing seems to be of limited value as an indicator for replacing amalgam restorations and predicting outcome [10]. Regardless of the results of the patch tests a regression of oral mucosal lesions after removal of amalgam has been found [23].

This raises the question of whether amalgam fillings of patients with OLP need to be removed? In a review the proportion of individuals achieving complete healing varied from 37 to 100% although, in total 15% of patients showed no improvement after replacement of their amalgam restorations [10, 21]. The disease course after replacement of amalgam is not uniform across the reported studies.

How can we identify lesions that would respond to amalgam replacement? A close topographical relationship between lesions and amalgam fillings appears to be the best predictor [8–11].

According to the grading of Thornhill, the strength of association between the mucosal lesions and the amalgam restoration is the key criterion. Only amalgam fillings in direct contact with the mucosa need to be removed to achieve lesion resolution [9]. Our results show that the grading did not differ between the presentation forms or the clinical form of OLP.

This leads to the question; which material can be recommended to the affected patients? According to Thornhill et al., the different replacement filling or crown materials used were equally effective. Inert materials are preferable [7].

Martin et al. defined risk factors for OLP: number of teeth with amalgam, total surfaces of amalgam, number of teeth with gold, corrosion, and bimetallism [24]. This is difficult to apply to our results, as the patients studied had fewer fillings, especially fewer amalgam fillings. The most common filling material in our study was composite.

Ahlgren et al. found a high incidence of contact allergy to gold in patients with OLP. The frequency of contact allergy to gold was 28.9% in patients with oral lichen lesions and 22.9% in the clinically examined control patients. They suggest dental gold to be part of the etiology or a maintenance factor for patients with oral lichen lesions [25]. Our investigation showed a correlation between gold and the generalized lichen form, which represents a more severe manifestation of OLP. We found no correlation with amalgam or metal.

The OHIP score of the 112 patients examined by us was more than 3 times higher than the average value of the German general population [18]. Numerous studies have examined the quality of life of patients with OLP. The OHIP scores were between 9.42 and 21.6 [26–31].

Patients with a reticular form of OLP had less pain and lower OHIP scores. We revealed in another study that patients with a reticular OLP had lower OHIP scores which implies a higher OHRQoL [32].

Our results show a high positive correlation between OHIP and pain. That means, the higher the pain, the higher the OHIP-value. Oral mucosal disease not only causes a local reaction but affects the whole patient. That means, pain severity is the most important contributor to the increase of OHIP-values, and severe pain is the most influential to increase OHIP-values.

A recent Cochrane review quoted that the impact of pain on physical, emotional, and social functions required multi-dimensional qualitative tools and health-related quality of life instruments that are uncommonly used in OLP trials [14].

One third of patients with OLP have psychological comorbidities like anxiety, depressive or distress symptoms [33–35]. It is believed that autoimmune diseases influence the psyche of affected patients. Interesting research by Pippi et al. investigated the influence of the clinical form of OLP on these psychological aspects. Patients with severe forms of OLP were not associated with certain psychological traits [36]. In our study patients with non-reticular OLP forms suffered more and had higher OHIP values.

Several external factors have been proposed to trigger OPL, including dental materials and psychological stress [37]. Stress is an important etiological factor that can trigger an attack of pain. So we have to not only treat the local reaction of the oral mucosa we have to treat the patient as a whole. Especially psychological factors need to be considered.

Early diagnosis and treatment of oral mucosal diseases can reduce the impact on the quality of life of affected patients in the future [38, 39].

### Limitations

Our sample included only patients from one dental clinic which limits generalization. The main limits are the reduced number of study subjects and not having a control group with patients after removal/replacement of dental restoration materials. So far, dental status, periodontal health, and oral hygiene have not been taken into account in our investigations. These factors additionally influence the OHRQoL and have to be regarded in the future.

## Conclusions

In summary, the OHIP scores were significantly higher in patients with OLP. We found no statistical differences in the clinical parameters between patients with amalgam and OLP lesions without metals. It is not necessary to replace amalgam fillings that are not in direct contact with mucosal surfaces. The individual factors of each patient are more important than the intraoral restorations.

## Supplementary Information

**Additional file 1.** OHIP-14 questionnaire.

## Data Availability

The datasets supporting the conclusions of this article are available at the Department of Cranio-Maxillofacial Surgery, University of Muenster, Germany. If someone wants to request the data from this study please contact the corresponding author.

## Abbreviations

OLP: Oral lichen planus
OHIP: Oral health impact profile
OHRQoL: Oral health related quality of life
OLR: Oral lichenoid reaction
