# Supplementary material for: Clinical characteristics of oral lichen planus and its causal context with dental restorative materials and oral health-related quality of life
Source: BMC Oral Health. 2021 May 15;21:262. doi: 10.1186/s12903-021-01622-z (PMC8126149; doi:10.1186/s12903-021-01622-z)
Supplement: Supplementary file 1 — Additional file 1. OHIP-14 questionnaire. [file 12903_2021_1622_MOESM1_ESM.docx]

Appendix

OHIP-14:

| **In the last month…** |
| --- |
| Have you had trouble pronouncing any words because of problems with your teeth, mouth, or dentures? |
| Have you felt your sense of taste has worsened because of problems with your teeth, mouth, or dentures? |
| Have you had painful aching in your mouth? |
| Have you found it uncomfortable to eat any foods because of problems with your teeth, mouth, or dentures? |
| Have you been self-conscious because of your teeth, mouth, or dentures? |
| Have you felt tense because of problems with your teeth, mouth, or dentures? |
| Has your diet been unsatisfactory because of problems with your teeth, mouth, or dentures? |
| Have you had to interrupt meals because of problems with your teeth, mouth, or dentures? |
| Have you found it difficult to relax because of problems with your teeth, mouth, or dentures? |
| Have you been a bit irritable with other people because of problems with your teeth, mouth, or dentures? |
| Have you had difficulty doing your usual jobs because of problems with your teeth, mouth, or dentures? |
| Have you felt that life in general was less satisfying because of problems with your teeth, mouth, or dentures? |
| Have you been totally unable to function because of problems with your teeth, mouth, or dentures? |
